# Space-directional approach to improve blood vessel visualization and temporal resolution in laser speckle contrast imaging

**DOI:** 10.1117/1.JBO.25.3.032009

**Published:** 2019-12-12

**Authors:** C. Elizabeth Perez-Corona, Hayde Peregrina-Barreto, Julio C. Ramirez-San-Juan

**Affiliations:** a Instituto Nacional de Astrofisica, Optica y Electronica (INAOE), Department of Optics, Puebla, Mexico; b Instituto Nacional de Astrofisica, Optica y Electronica (INAOE), Department of Computer Sciences, Puebla, Mexico

**Keywords:** blood vessels, digital processing images, laser speckle contrast imaging, speckle

## Abstract

Blood flow is a parameter used to diagnose vascular diseases based on flow speed, blood pressure, and vessel size. Different techniques have been developed to estimate the relative blood flow speed and to improve the visualization of deep blood vessels; one such technique is laser speckle contrast imaging (LSCI). LSCI images contain a high level of noise mainly when deep blood vessels are imaged. To improve their visualization, several approaches for contrast computation have been developed. However, there is a compromise between noise attenuation and temporal resolution. On the one hand, spatial approaches have low spatial resolution, high temporal resolution, and significant noise attenuation, while temporal approaches have the opposite. A recent approach combines a temporal base with a directional process that allows improving the visualization of blood vessels. Nevertheless, it still contains a high level of noise and requires a high number of raw frames for its base. We propose, a space-directional approach focused on improving noise attenuation and temporal resolution for contrast computation. The results of reference approaches and the proposed one are compared quantitatively. Moreover, it is shown that the visualization of blood vessels in LSCI images can be improved by a general morphological process when the noise level is reduced.

## 1 Introduction

Blood flow carries oxygen and nutrients through the blood cells to keep tissues healthy.^1^ As the blood flow travels through all the organs of the body, it is possible to identify some diseases because sometimes the disease affects the behavior of the blood flow. For example, in a port-wine stains laser treatment, it is important to distinguish to what extent the blood vessels are destroyed after receiving the treatment, to establish its effectiveness.^2^ In general, blood vessel visualization and blood flow estimation in the skin are important issues in the medical field.

Laser speckle contrast imaging (LSCI) is a noninvasive technique developed in the 1980s for blood perfusion assessment.^3^ This method is based on obtaining a set of raw speckle images (RSIs) and computing a contrast representation of them, which allows the visualization of blood vessels and an estimation of a relative flow index in tissues, such as skin,^2^ brain,^4^ and retina.^5^ To obtain the contrast representation, the LSCI method is used, for which several approaches have been reported in the literature. Spatial approaches, such as spatial LSCI (sK)^1^ and spatial-average LSCI ($s_{\mathrm{avg}}K$),^6^ use a sliding analysis window, where the contrast is computed from the pixels inside the window until a single contrast image is generated or the average contrast is computed for a set of images, respectively. Temporal approaches, such as temporal LSCI (tK),^5^ take into account a single pixel through the temporal dimension (m frames) and compute the contrast from the temporal values of that pixel. A combination of both approaches, namely spatial–temporal LSCI (stK),^7^ can also be used. The main difference between these approaches consists in the selection of the set of pixels used for contrast computation.

The main drawback when LSCI images of a deep blood vessel are analyzed is the high noise level due to so highly scattering coefficient of the epidermis (or skin) on the top of the blood vessel. Even when contrast improves blood vessel visualization in the LSCI images, the best results are obtained in superficial blood vessels. Spatial approaches provide a high noise attenuation, but at the cost of low spatial resolution, and vice versa for the temporal contrast. Recently, another kind of approach based on variation detection in the pixel values has emerged to try to deal with noise attenuation, without significantly reducing temporal resolution.^8^ However, the noise level is still a drawback at larger depths (>300  μm).^9^

In this work, a method to improve the visualization of blood vessels is proposed, which not only deals with noise attenuation but also maintains a high temporal resolution. This proposal is based on the variation detection of pixel values and digital image processing techniques. Thus, the contrast is computed adaptively, allowing noise reduction in the final image. Moreover, this method is complemented with a morphological process to highlight the vessel region. A preliminary version of this work has been reported by Perez-Corona et al.^10^

## 2 Basic Concepts

### 2.1 Experimental Setup

#### 2.1.1 Skin phantom

**Fig. 1:**
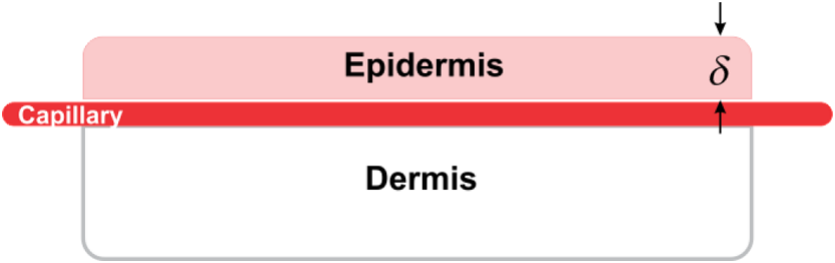
Skin phantom. In order to simulate a deep blood vessel, the epidermis thickness layer was δ=0,200,400, and 500  μm.

In this work, we used a skin phantom (Fig. 1) with absorption and scattering properties similar to the skin.^11^ The dermis was prepared with a solid resin, whereas polydimethylsiloxane and titanium dioxide powder were used for the epidermis, both at the proper concentration. Prior to the dermis solidification, a glass capillary (inner diameter 700  μm) was placed on top to simulate a straight vessel. Different epidermis thickness layers (δ=0 to 500  μm) were made and placed on top of the dermis to simulate the vessel depth.

#### 2.1.2 LSCI setup

**Fig. 2:**
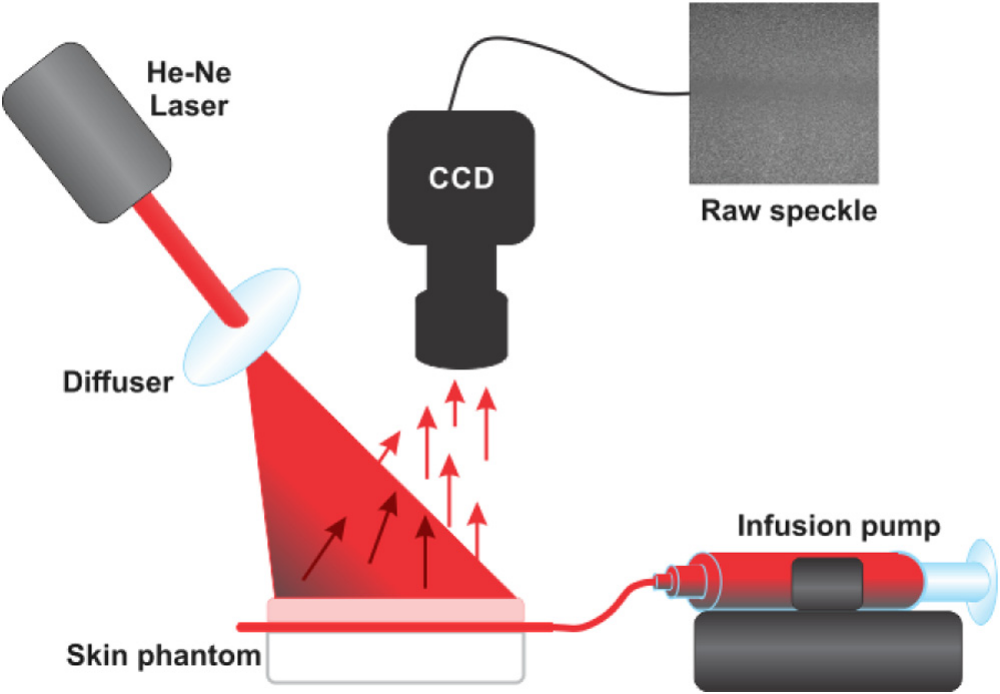
LSI experimental setup: a He–Ne laser (632.8 nm) illuminates the skin phantom through a diffuser for homogeneous illumination, the CDD camera equipped with a macro lens acquires a sequence of 30 RSIs with an exposure time of T=10  ms.

Figure 2 shows the experimental setup used in this work. A coherent light source (He–Ne laser, λ=632.8  nm) illuminates homogeneously the skin phantom through a diffuser (Model ED1-C20, Thorlabs, Inc.). The backscattered light coming from the sample is captured by a CDD camera (Model Retiga 2000R, QImaging, Canada) and a lens (NAV ITAR ZOOM 7000 with f/#=2.5, focal length = 18 to 108 mm, and a working distance of 5″) with an exposure time (T) of 10 ms, and the speckle size of ≈7.9  μm. In the capillary, an intralipid 1% water mix circulates as a blood substitute.^11^ This is injected by a syringe and an infusion pump (Model NE-500, New Era Pump Systems, Inc.) controls the injection speed of the liquid.^12^

### 2.2 Contrast Calculation

**Fig. 3:**
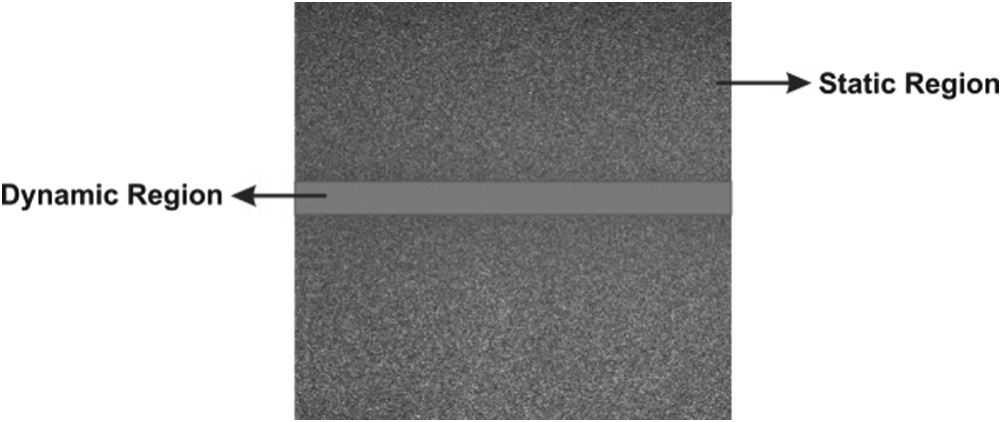
Example of a raw speckle image.

In an RSI from biological tissue, we can identify two kinds of regions (Fig. 3): the “static region,” which corresponds to the “static” tissue (skin) and the “dynamic region,” which corresponds to the blood flow. The static region is observed for pixels in a wide range of gray levels, whereas the dynamic region is observed in gray levels with closer values having a blurring appearance. A blurred region is generated when there are particles in movement, in this case, the blood cells, and this is related to the dynamic region and blood vessels.^13^

Contrast is defined as follows:

$$K=\frac{\sigma }{\left\langle I\right\rangle },$$ (1)

where K is the contrast, σ is the standard deviation, and ⟨I⟩ is the mean value of light intensity. Contrast allows describing the gray level variation that exists around each pixel. If such variation is high, as in the static region, the K value approaches 1; otherwise, the closer the values are, the closer K will be 0. Thus, the blood vessel regions are those with the lowest values of K.

Several algorithms have been used for contrast computing. The spatial algorithm (sK) uses a sliding window of W×W pixels. Commonly, W takes values of 5 or 7. Using Eq. (2), the contrast value associated with the (i,j) pixel of the RSI (I) is computed from the values belonging to its spatial neighborhood (sliding window):^1^

$$K_{i,j,t}=\frac{\sqrt{\frac{1}{m^{2}}\overset{i+\frac{m-1}{2}}{\underset{x=i-\frac{m-1}{2}}{\sum }}\overset{j+\frac{m-1}{2}}{\underset{y=j-\frac{m-1}{2}}{\sum }}{\left(I_{x,y,t}-\mu_{i,j,t}^{s}\right)}^{2}}}{\mu_{i,j,t}^{s}},$$ (2)

$$\mu_{i,j,t}^{s}=\frac{1}{m^{2}}\sum_{x=i-\frac{m-1}{2}}^{i+\frac{m-1}{2}}\sum_{y=j-\frac{m-1}{2}}^{j+\frac{m-1}{2}}I(x,y,t),$$ (3)

where t is the t’th frame of a set of m RSIs and $\mu_{i,j,t}^{s}$ is the mean intensity in the current analysis window.^9^^,^^14^ This process is applied to all the frames of the package. Thus, the spatial contrast image is computed as follows:

$$sK_{i,j}=\frac{1}{m}\overset{m}{\underset{t=1}{\sum }}K_{i,j,t}.$$ (4)

**Fig. 4:**
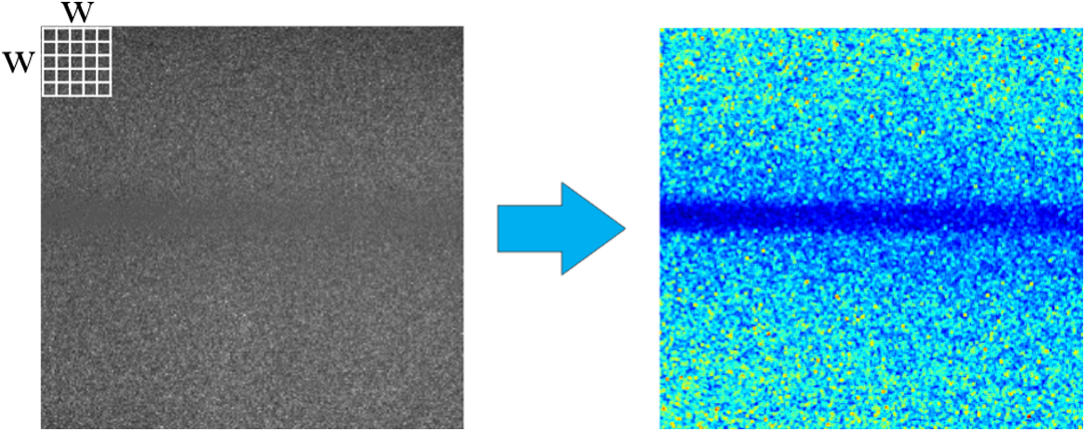
Spatial contrast algorithm (sK): an RSI (left), where the pixels within the white grid (sliding window) are used to calculate the contrast of the central pixel and to obtain the sK image (right).

Figure 4 shows how the RSI is processed. The analysis window (white grid) slides pixel-by-pixel, computing the contrast value of the central pixel by taking into account the $W^{2}-1$ pixels around it. The final sK image shows a higher difference in value between the static and the dynamic region, which improves the visualization of the blood vessel region. The main advantage of this approach is that it achieves a high noise attenuation; however, it also has a low spatial resolution since the final image is an average of the m spatial contrast images. When m sK images are averaged, the resulting image is called spatial average contrast image ($s_{\mathrm{avg}}K$).^1^

**Fig. 5:**
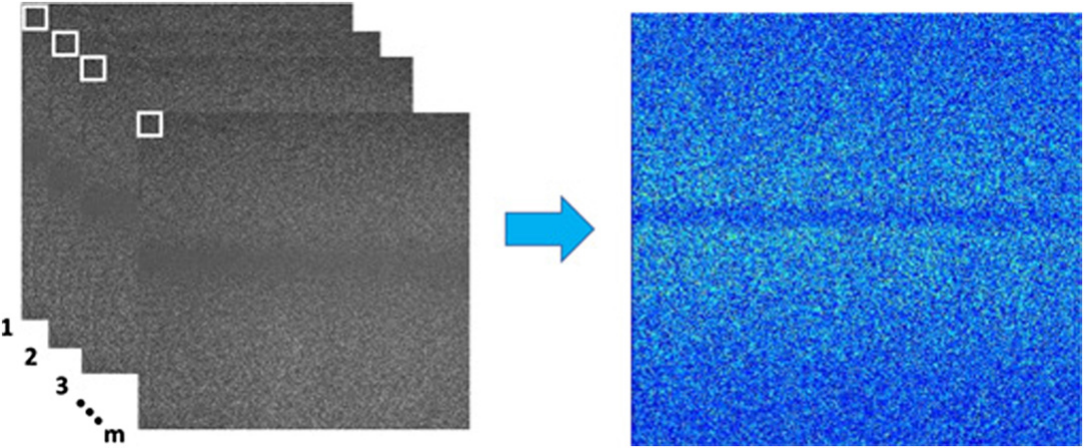
Temporal contrast algorithm (tK): set of RSIs (left), where the white pixels are used to calculate the temporal contrast for each pixel in order to obtain the tK image (right).

Temporal contrast (tK) computes the m consecutive RSIs by taking the same pixel from each image in the temporal sequence; then, the temporal analysis window has dimensions 1×m. Thus, tK is expressed as^9^

$$tK_{x,y,l}=\frac{\sqrt{\frac{1}{m}\overset{l+m/2}{\underset{t=l-m/2}{\sum }}{\left(I_{x,y,t}-\mu_{x,y,l}^{t}\right)}^{2}}}{\mu_{x,y,l}^{t}},$$ (5)

where

$$\mu_{x,y,l}^{t}=\frac{1}{m}\sum_{t=l-m/2}^{l+m/2}I_{x,y,t},$$ (6)

where l is the central frame of the set, (x,y,l) is the central element in the temporal window, $\mu_{x,y,l}^{t}$ is the temporal mean, and tK is the image of temporal contrast. Unlike spatial contrast, temporal contrast provides a higher spatial resolution, but the noise level remains high^5^ in the final contrast image, as shown in Fig. 5. Accordingly, with Vaz et al.,^9^ in this method, it is necessary to analyze from 3 to 30 pixels to keep a good resolution in the contrast image; for practical applications, the mean number of frame is 15.^9^

**Fig. 6:**
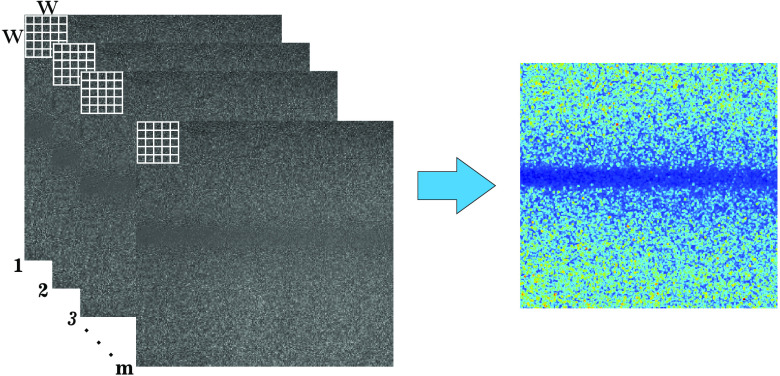
Spatiotemporal contrast algorithm (stK): the cube of W×W×m pixels, taken from a set of RSIs (left), is used to calculate the contrast of the central pixel in order to obtain the stK image (right).

Spatiotemporal contrast (stK)^7^ uses an analysis window of W×W×m, where W is the window size (commonly 5×5) and m is the number of frames to analyze (Fig. 6) so that a cube is generated to calculate the contrast. The spatiotemporal approach tries to combine the advantages of the two previous algorithms.

**Fig. 7:**
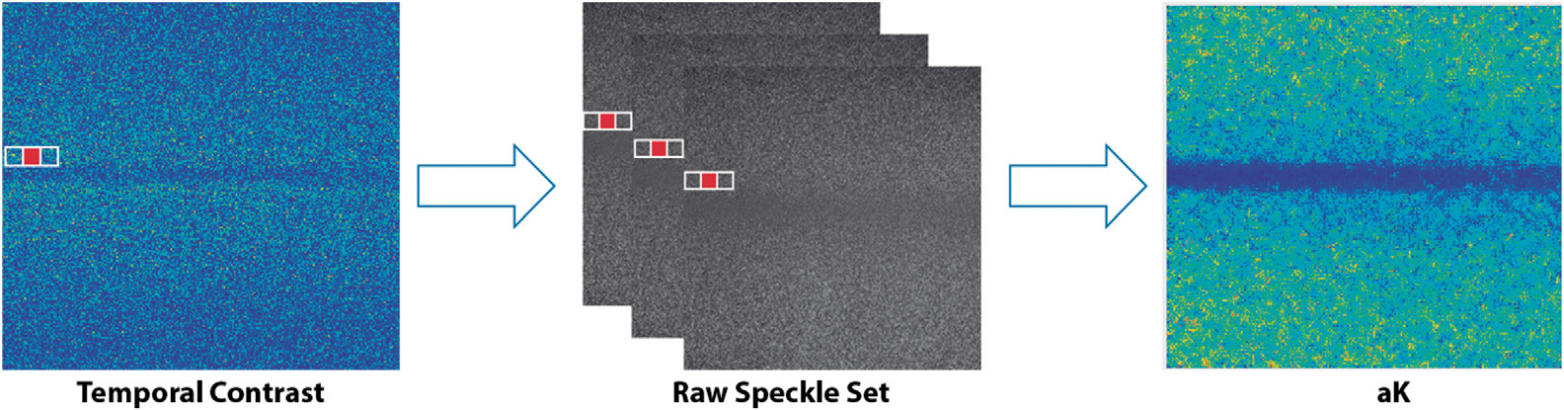
Anisotropic contrast algorithm (aK): (left) temporal contrast image, where an l direction is selected (e.g., 0 deg) from a tK image for (middle) new contrast computation through the $N_{F}$ frames from the RSIs and (right) the resulting aK image.

Anisotropic contrast (aK) is a recent algorithm that computes the contrast, preferentially along with the blood flow, by considering one direction that is selected based on the minimal contrast gradient with respect to a central pixel ($P_{0}$) using Eq. (7). The gradient is computed from a temporal contrast image and, once the direction (l) has been selected, the contrast is recalculated from the pixels that lie in the line with l direction through a set of $N_{F}$ RSIs (Fig. 7).^8^ This approach is able to provide noise attenuation and to maintain a high temporal resolution since it only uses three frames:

$$\arg[l_{0}]=\underset{l\in 0^{{}^{\circ}}\to 180^{{}^{\circ}}}{\arg\;\min}\;[\underset{P\in l}{\sum }{\left(K_{P}-K_{P_{0}}\right)}^{2}],$$ (7)

where arg min is the argument minimum, $P_{0}$ is the central pixel, and l comprises the set of possible analysis directions.

### 2.3 Contrast-to-Noise Ratio

Contrast-to-noise ratio (CNR) provides information about image quality in noise terms. In general, it provides information about how different two region of interest (ROIs) are,^15^ i.e., it measures the contrast degradation. In this case, the measurement involves the difference between the region of the vessel and the region of the tissue. CNR is defined as^16^

$$\mathrm{CNR}=\frac{\mu_{\text{vessels}}-\mu_{\text{tissue}}}{\sigma_{\text{tissue}}},$$ (8)

where $\mu_{\text{vessels}}$ and $\mu_{\text{tissue}}$ are mean contrast values of vessel and tissue regions, respectively, and $\sigma_{\text{tissue}}$ is the standard deviation of the tissue, i.e., the variation of contrast in the background. When CNR is high, it is because the contrast between the dynamic and the static regions is also high. This is a desirable characteristic in the final contrast image since it makes reference to a high distance in value between the two main regions, which is associated with a better visual distinction.

## 3 Space-Directional Contrast

**Fig. 8:**
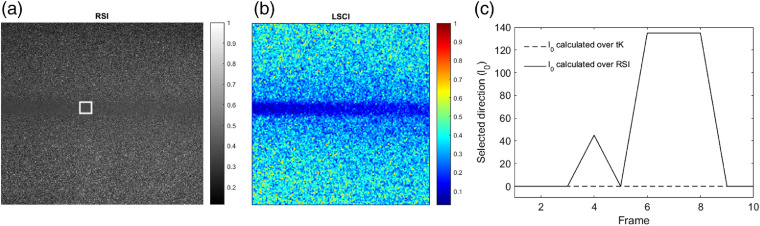
(a), (b) Comparison of RSI and LSCI images. (c) Nonconstant behavior of the direction with minimum variation analyzed over the dynamic region (white square) from RSI set and its comparison with an assumed constant direction estimated from tK.

Previous approaches for contrast representation of LSCI images have a compromise between noise attenuation and temporal resolution. Both of them are desirable characteristics; the former is necessary for an accurate blood vessel visualization, whereas the latter is important for further processes, such as speckle flow index (SFI) calculation. For this reason, it is necessary to develop approaches that focus on both characteristics. The proposed method maintains a high temporal resolution and attenuates noise. In order to do this, a set of RSIs is analyzed; these images were obtained with the experimental setup, as explained in Sec. 2.1. A set of 30 images was obtained for each depth and velocity value. This approach assumes that the base (raw set) contains ROIs (blood vessels) that are imperceptible due to an intense noise in the image [Figs. 8(a) and 8(b)]. For explanatory purposes of this article, the high-intensity variations are considered noise and are associated with the static speckle coming from the surrounding of the blood vessel. It is expected that the dynamic region has low-intensity variations due to blood flow; then, high variations over the dynamic region are considered as noise. The deeper the blood vessel, the noisier the LSI image since the thickness of the tissue increases. For the study of LSCI images, noise does not provide relevant information and, therefore, it must be attenuated to improve the visualization of blood vessels.

Contrast computation helps to attenuate the noise by taking into account surrounding information (analysis window) that generates wider regions of similar value and allows the visualization [Fig. 8(b)]. Additionally, a selection that avoids taking into account noise pixels in contrast computation could provide an improved LSCI image. An approach as aK makes a selective process by estimating an optimum direction $l_{0}$ [Eq. (7)]. Since the base image (tK) already contains attenuated noise values, as a result of processing n=30 RSIs, the direction $l_{0}$ is representative of the RSI set used for calculating tK. However, a subset of frames (≪n) is used for final aK image and then, $l_{0}$ may not represent the minimum gradient of that subset. For example, consider the ROI (white square) in Fig. 8(b) that corresponds to the dynamic region, whose direction $l_{0}$ has been estimated over a tK image (dotted line). If the same criterion is followed for estimating $l_{0}$ frame-by-frame from an RSI subset (continuous line), then it can be observed that the direction with less variation from tK does not necessarily represent the direction with less variation from the RSI.

**Fig. 9:**
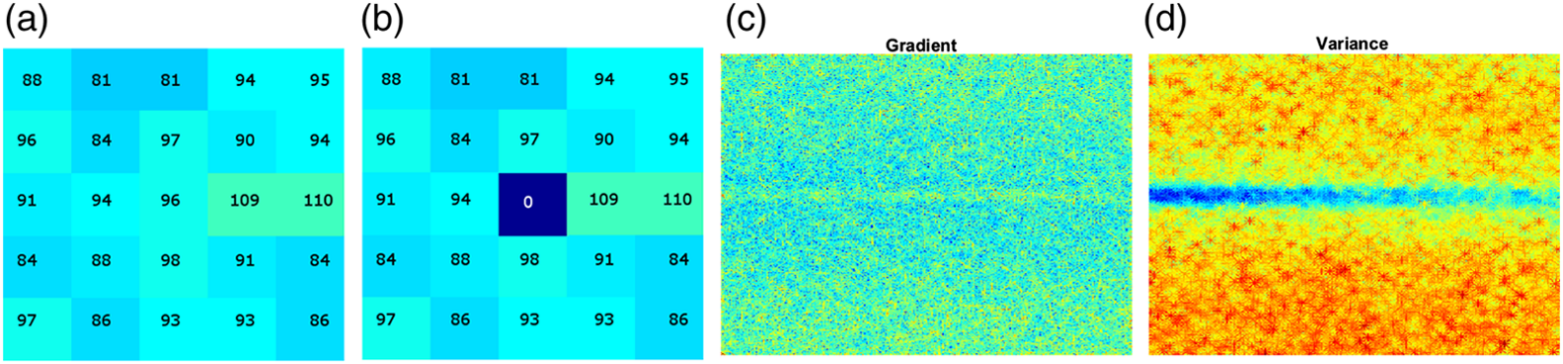
Example of an analysis window (a) when the central pixel is representative of the region and (b) when is a noise pixel (outlier); images generated from (c) gradient and (d) variance.

Moreover, the criterion selection is also a factor that influences the noise level in the contrast image. In order to select the direction that can provide the most accurate contrast value, it is necessary to establish a reference value so that the criterion can measure how similar or different are the values in a certain direction with respect to the reference. For example, consider a random ROI over the dynamic region [Fig. 9(a)] and a criterion as mean square error for measuring the minimum variation. If the central pixel (96) is taken as reference, the minimum variation occurs at 90 deg since the values in that direction are closer to the central one. However, if the central pixel has an outlier value [Fig. 9(b)], e.g., a noise pixel 0, the minimum variation occurs at 135 deg because now the values in that direction are closer to the central one. As the contrast value depends on the pixels that are in the suggested direction, the contrast will be low for the case of Fig. 9(b) but high in the case of Fig. 9(a), and the latter will generate again a noise pixel in the dynamic region; the effect will increase as the blood vessel becomes deeper (noisier). Instead, if the reference is a representative value of the neighborhood, as a central trend measure, the noise effect caused by outliers could be attenuated by improving the quality of the contrast image. The general effect can be observed in images generated from the gradient and variance [Figs. 9(c) and 9(d)].

**Fig. 10:**
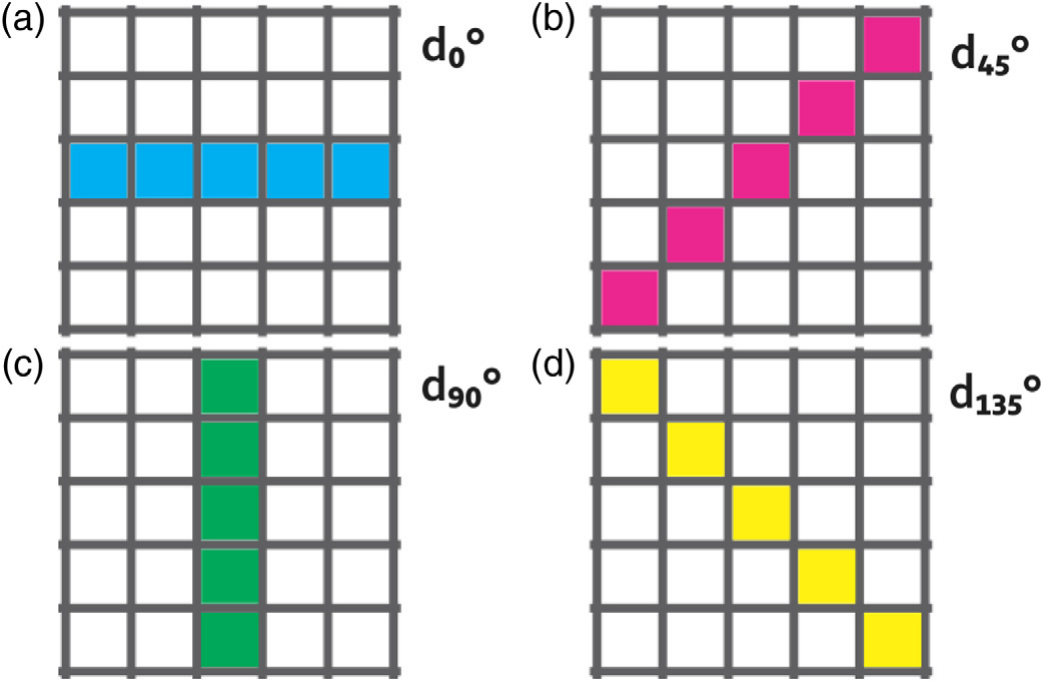
Four directions considered by the analysis window. (a) $d_{0\;\deg}$, (b) $d_{45\;\deg}$, (c) $d_{90\;\deg}$, and (d) $d_{135\;\deg}$.

**Fig. 11:**
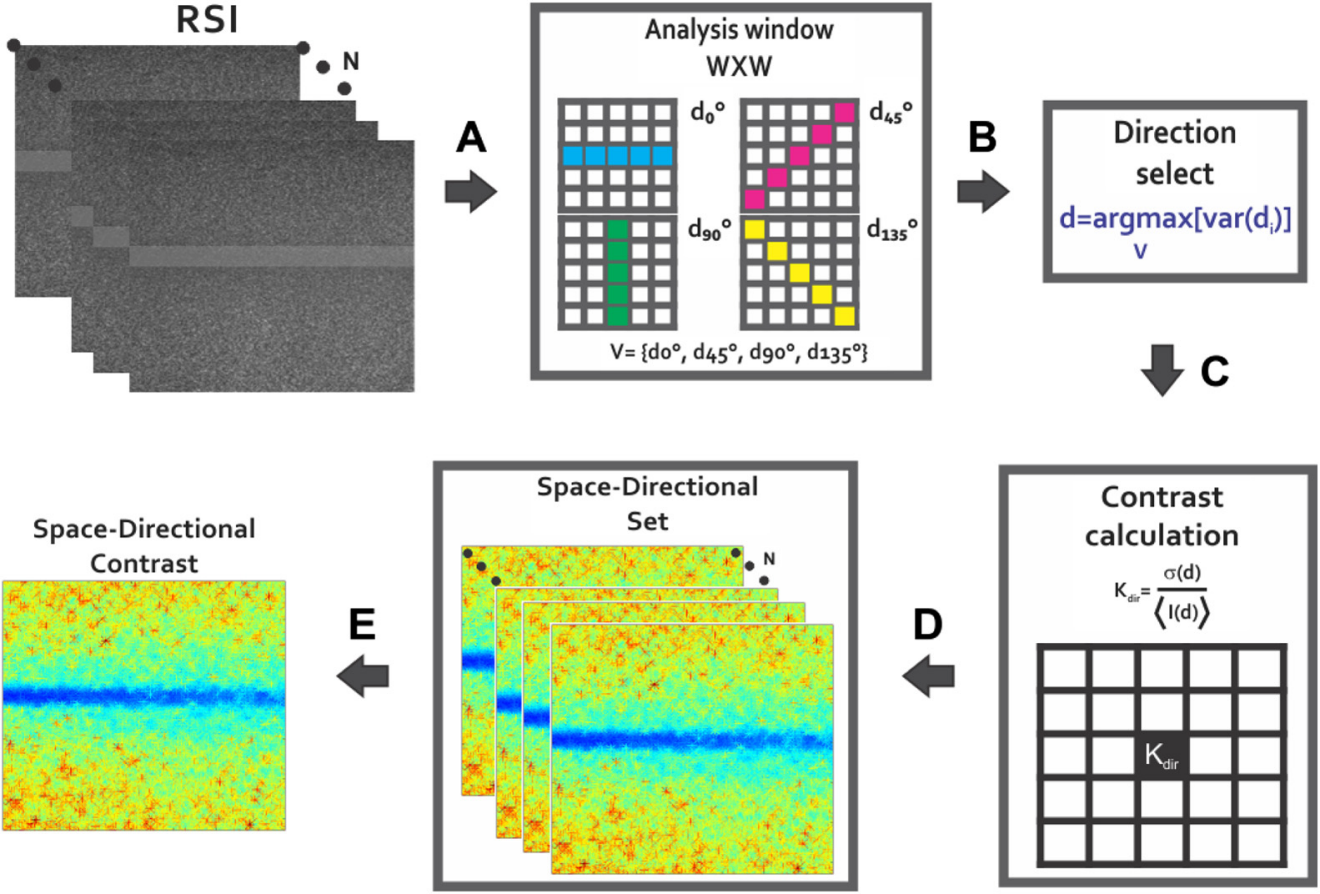
Proposed space-directional contrast approach for contrast calculation: from each RSI of the set (A) the variance value is calculated in four directions, (B) the direction d is selected by the maximum variance criterion, (C) contrast is calculated at d direction of the analysis window pixel by pixel for obtaining (D) a set of space-directional images (E) that are averaged for provide the final contrast image sdK.

Thus, it proposed a directional analysis that takes into account the next: (i) an estimation direction per frame and from the RSI data and (ii) a criterion based on variance for measuring the intensity changes due to which it uses the mean intensity as a reference for the rest of the values and is less affected by outliers. Moreover, to take advantage of the high variations in intensity, the next hypothesis is proposed: if the contrast is computed by taking the direction with maximum variation, the static region will increase its contrast value compared to the traditional contrast calculation, whereas the contrast in the dynamic region will have a small change since most of its values have a high similitude. In this way, the difference in contrast between both regions will be increased, improving the dynamic region visualization. Thus, the raw LSCI images are analyzed under the following criterion:

$$d=\underset{V}{\arg\;\max}[\mathrm{var}(d_{i})],$$ (9)

where $V=\{d_{0\;\deg},d_{45\;\deg},d_{90\;\deg},d_{135\;\deg}\}$ is a set of vectors of length 1×W that corresponds to (0 deg, 45 deg, 90 deg, and 135 deg), the four main directions within the analysis window (Fig. 10); the variance of the vectors in V is var; and d corresponds to the index associated to the vector with maximum variance. As previously explained, since the values are closer inside the dynamic region, it is then expected that the computed contrast will be representative of the region even in the direction with higher variance. The direction estimation is computed for each pixel of the raw images. Once the direction d is known for the pixel I(i,j), the pixels belonging to the vector $v_{d}$ are used to compute contrast value K(i,j). This process is applied to all the raw LSCI images (avoiding the assumption that the direction of maximum variation remains stable along with all the frames) so that a new set of spatial contrast images that are calculated based on directional criterion is obtained. As in the traditional spatial contrast, all the images are averaged at the end. This way, the final result of the proposed space-directional approach is obtained (Fig. 11).

## 4 Results and Discussion

### 4.1 Space-Directional Images

In this work, an *in-vitro* straight vessel at different depths was processed with this methodology. For each depth, a set of 30 RSIs was obtained. Images have a size of 334×349  pixels and were processed with the proposed algorithm and with the algorithms described in Sec. 2.2. Since contrast is a statistic measure, the number of elements taken into account for contrast estimation is related with a better contrast estimation.^9^ Therefore, the RSIs were processed under the same conditions, i.e., by considering the same number of pixels, in order to provide a fair comparison among the different approaches (Table 1). Also, in order to make a direct comparison with the closest approach aK, a spatial window of 9×1 was taken as a reference. Since one of the objectives of this proposal is to improve the temporal resolution, several tests were performed in order to identify how the results are affected by the number of frames used in contrast computation using sdK. The tests demonstrated that the change in CNR values is not significant after three frames, i.e., the difference between 3 and 30 frames was about 0.038.

**Table 1:** Parameters used to compute contrast comparison.

Approach	W×W	Frames
$s_{\mathrm{avg}}K$	3×3	3
stK	3×3	3
aK	9×1	3
sdK	9×1	3

**Fig. 12:**
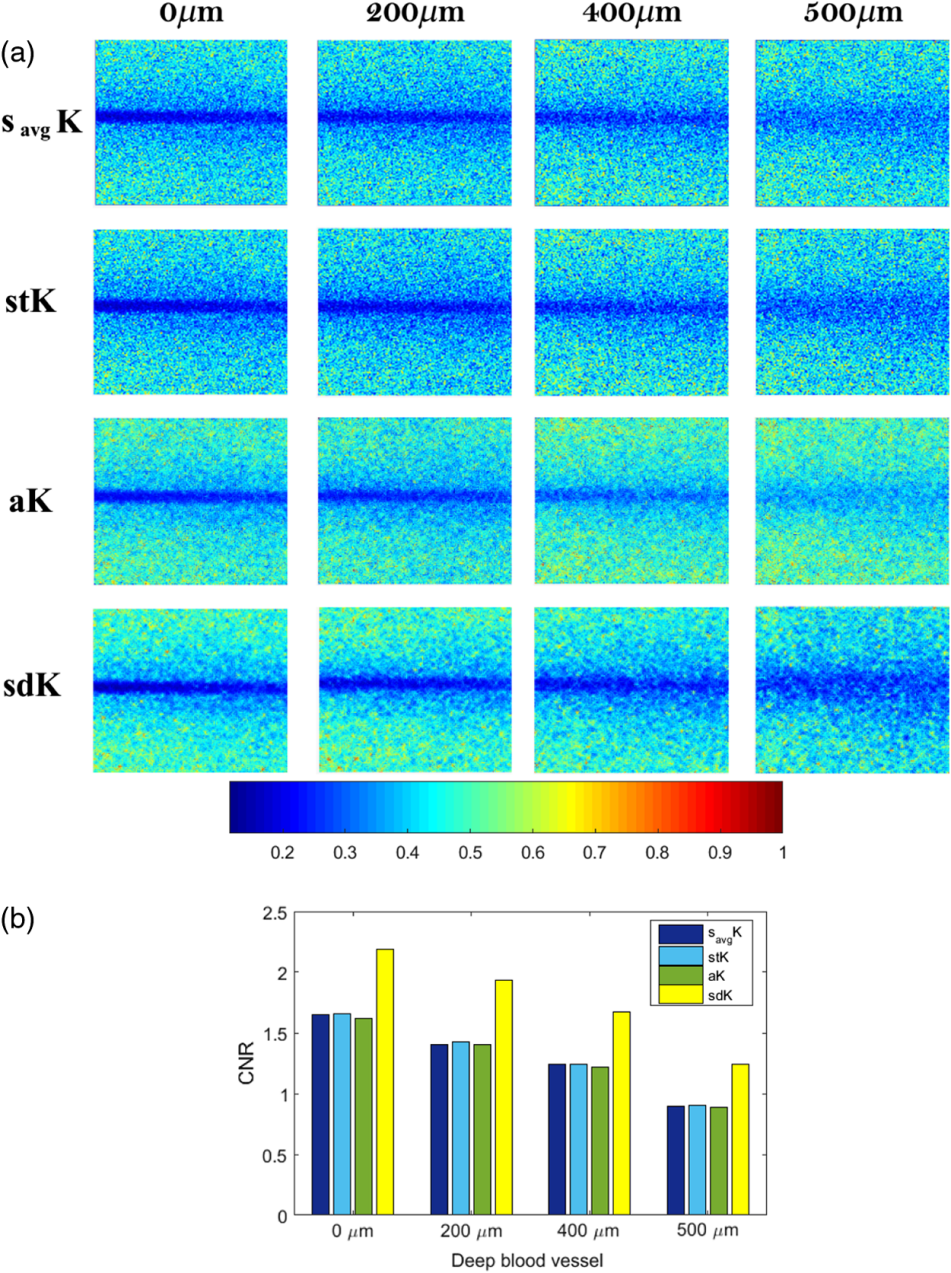
(a) Contrast images computed with different LSCI approaches and at different depths and (b) their corresponding CNR measures.

The first comparison is with a superficial vessel at 0  μm depth, where the difference between the static and the dynamic region is easy to distinguish with all comparative LSCI approaches (Fig. 12). One of the main characteristics of $s_{\mathrm{avg}}K$ is an improvement of noise attenuation since it takes into account neighborhood information [first row of Fig. 12(a)]. However, the literature establishes that the best performance of $s_{\mathrm{avg}}K$ is obtained by processing at least 15 RSIs.^9^ In this case, $s_{\text{avg}}K$ was processed with only three frames in order to test its performance and compare it under the same conditions of the other approaches. As can be seen in the results, $s_{\mathrm{avg}}K$ attenuates noise level while also it defines the dynamic region; the blood vessel definition is possible even at 400  μm.

$s_{\mathrm{avg}}K$ and stK show greater similarity in improved noise attenuation. Nevertheless, in stK, the dynamic region keeps higher connectivity, in the sense that it is perceived as a single region, even when depth increases as observed at 400 and 500  μm in Fig. 12(a) (second row). This is an important characteristic because if a region is disconnected (divided), then its visual perception and interpretation change. Consider, for example, an *in-vivo* image that contains curved contiguous blood vessels; if a single blood vessel is disconnected in two points, then it may be perceived as three blood vessels or as a shorter vessel that does not reach a certain region, even as a bifurcation of another one. Then, connectivity is a desirable characteristic in contrast approaches. The anisotropic method (aK) tries to maintain a high temporal resolution while it reduces the noise by selecting one direction to calculate the contrast. The results achieve a significant improvement, with respect to the two previous, that is observed in the blood vessel definition [third row of Fig. 12(a)]. High temporal resolution is reached by processing only three frames. However, a high noise level still remains in the resulting images. This is because of aK uses as a baseline image of temporal contrast, which contains high-level noise, for selecting the direction l, where the minimum gradient occurs, i.e., the selection takes into account the integrated information temporarily. Then, it is assumed that the l direction is maintained also spatially during the $N_{F}$ raw frames and the aK value is recalculated with such information. This generates that, although improvement is achieved, the resulting image still presents a high noise level. Moreover, even when aK reaches an improvement with only three frames (in the spatial process), it still depends on the information provided by tK, in which the performance requires at least 15 frames.^9^

**Fig. 13:**
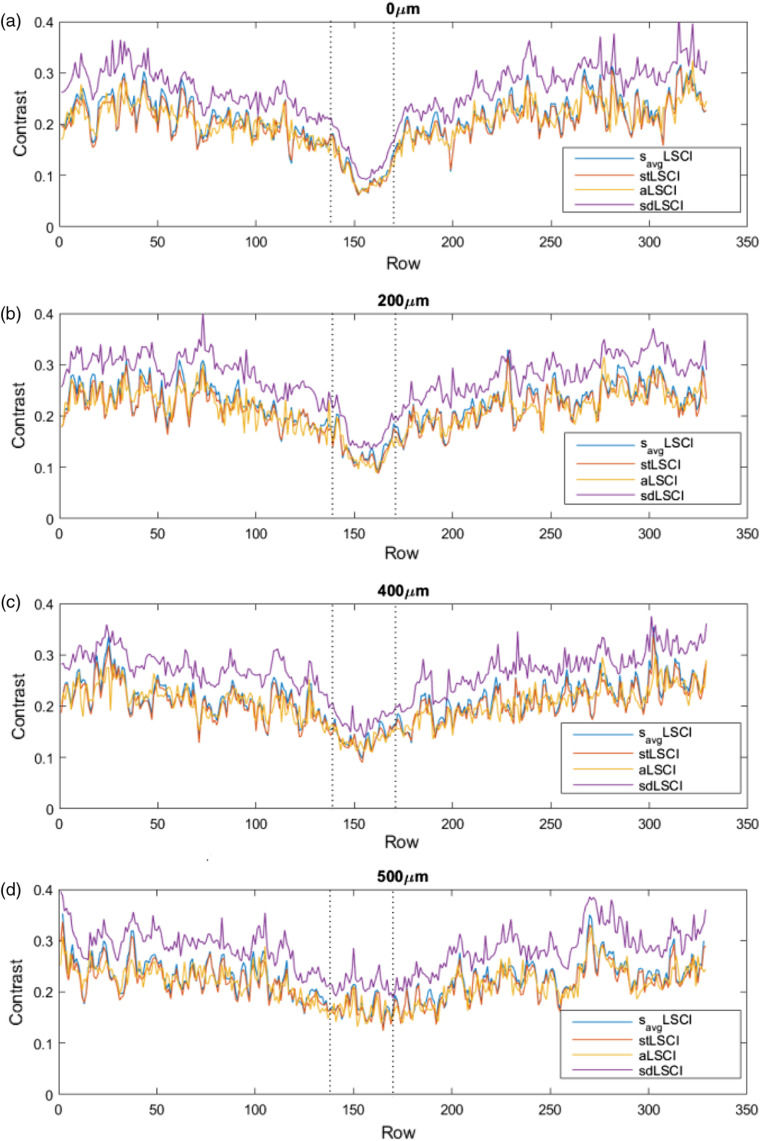
Unidimensional profiles of the blood vessel region in Fig. 12(a). (a) 0  μm, (b) 200  μm, (c) 400  μm, and (d) 500  μm.

On the other hand, sdK takes into account the variation in value that occurs from one frame to another. sdK takes as baseline raw images to guarantee that the contrast values are computed from the original information and avoid the dependence and influence of another approach. Moreover, the direction selection is done individually in each frame allowing to contribute with the best selection of pixels according to the established criterion. With a contrast computation based on a spatial processing and a selective direction, a significant noise attenuation is reached by sdK, as shown in Fig. 12(a). The used criterion allows a higher contrast between regions, which improves blood vessel definition. In order to provide a quantitative evaluation of the results obtained with the described approaches, the respective CNR values were calculated for 0, 200, 400, and 500  μm depths [Fig. 12(b)]. Also, Fig. 13 shows graphically the distance between the static and the dynamic region reached by the approaches at different depths, where the inverted peak, indicated between vertical dotted lines, is associated to the blood vessel location. The proposed sdK reaches an improvement by processing three frames, as stated in Table 1.

A predefined binary mask was used to isolate the dynamic and static regions and to calculate their respective statistics according to Eq. (8); the mask was generated by manually segmenting the dynamic region. Since the images correspond to *in-vitro* tests, the capillary (associated with the blood vessel) has the same image location at the different depths and location is known. The CNR value indicates the difference in contrast between dynamic and static regions, i.e., at a higher value, a better visualization. As observed in Fig. 12(b), sdK enhances blood vessel visualization by improving noise attenuation and this behavior remains at higher depths.

**Fig. 14:**
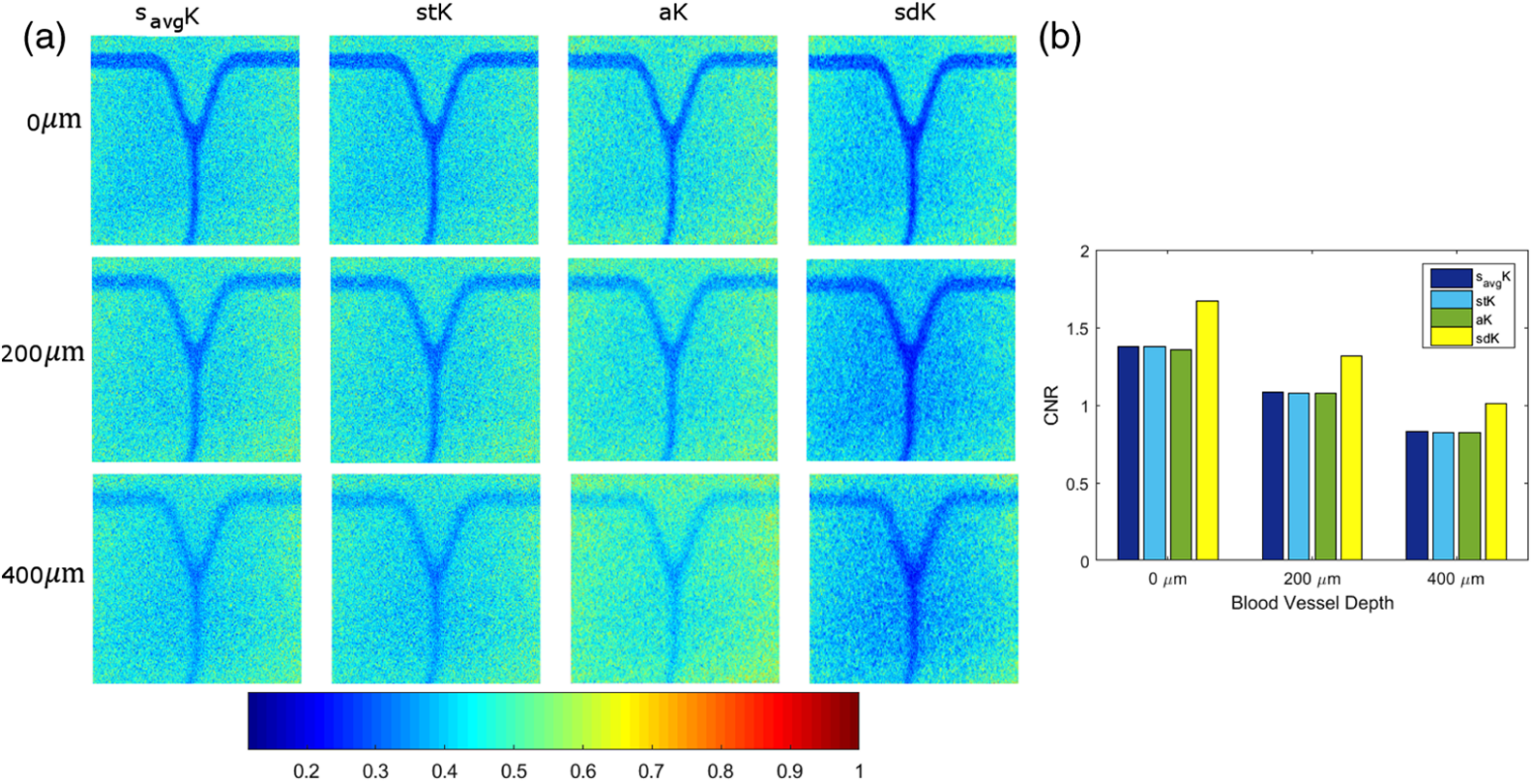
(a) Comparison of the performance of different approaches when the dynamic region changes its orientation and (b) their respective CNR measures.

In addition, the proposed sdK approach was also tested in *in-vitro* blood vessels with different morphology (bifurcated blood vessels) in order to know its performance when the orientation changes; this new set of images was also acquired using the experimental setup, as described in Sec. 2.1. Three tests were done at 0, 200, and 400  μm, as shown in Fig. 14(a). As can be seen, the approaches that taken into account the directional information, aK and sdK, provide an improvement in blood vessel visualization and temporal resolution (results were obtained with the same parameters of Table 1). It is observed that sdK reaches a higher contrast between the static and dynamic regions and keeps stronger connectivity inside the dynamic region, which makes it more robust to the noise generated by the blood vessel depth. This is also reflected in the CNR measures [Fig. 14(b)] that, as in previous tests, reach higher values with respect to other contrast approaches and at different depths. Besides, it is demonstrated that, although only four discrete directions are considered, sdK is not affected by changes in direction and is able to provide a significant improvement with respect to the other approaches.

**Fig. 15:**
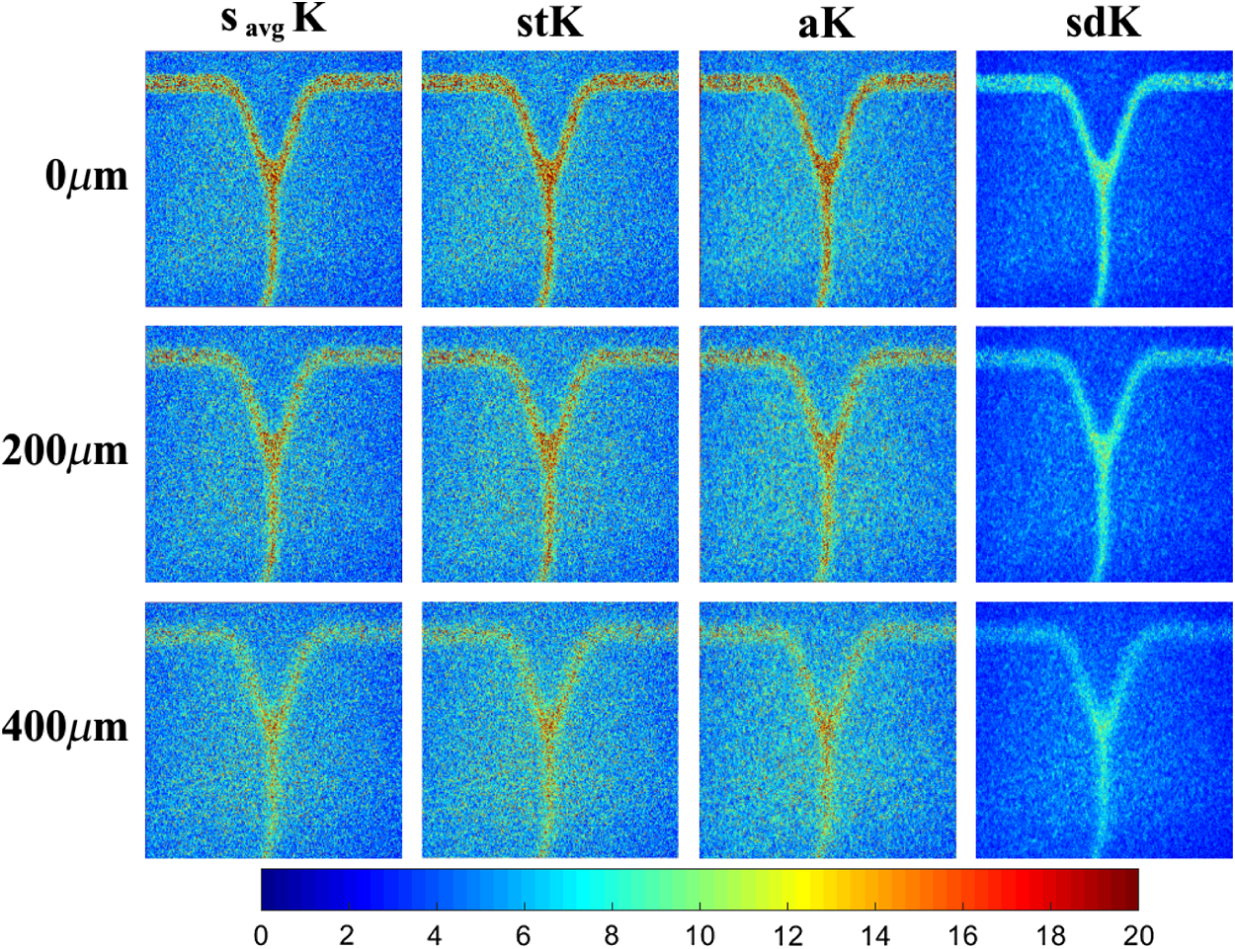
Comparison of the results of Fig. 14 in a representation of SFI.

Figure 15 shows the SFI map (flow speed $\propto \mathrm{SFI}=1/K^{2}$) for the different approaches at 0, 200, and 400  μm. As we can see, the noise around the blood vessels is attenuated, allowing a better definition and visualization of them. In this case, the SFI images do not have the purpose of measure, the relative blood flow. The computing time of the four approaches compared in Fig. 12 was measured taking into account the parameters of Table 1 and an image size of 334×349. The algorithms were implemented in MATLAB running on a PC with an Intel Core i7 at 3.6 GHz and 16 GB RAM. $s_{\mathrm{avg}}K$ and stK have lower processing time of 1.6 and 0.9 s, respectively. For aK and sdK, it is expected that the processing time increases since the contrast computation is not direct but it involves a directional analysis. Thus, the contrast processing took 9.3 s for aK and 1.4 s for sdK. The main difference in processing time is because sdK does not require a contrast recomputing after the directional analysis.

### 4.2 Space-Directional Images Improvement by Erosion

A complementary step is proposed to improve blood vessel definition and connection. This step consists of applying a morphological erosion to the resulting sdK image. Morphological operations are common in image processing to improve the definition of the structures in the image. In morphological operations, the operators work with two sets: the image processed (active image) and the structuring element (SE), that is, a kernel with a shape that encompasses the set of pixels of the active image to be considered by the operator. The active image can be modified by the different shapes or sizes of the SE.^17^ The morphological erosion (⊖) allows the central pixel to take the minimum value of its neighborhood as follows:

$$(I⊖\mathrm{SE})(x,y)=\underset{(i,j)\in SE}{\min}\{f(x+i,y+j)\},$$ (10)

where (x,y) are the coordinates of the central pixel in the image and (i,j) are the displacements, with respect to (x,y), that involve the pixels in the SE neighborhood, as explained by Sagar.^18^

In this way, the eroded image enhances the smaller values, generating a darker image in terms of gray levels. Regarding contrast values, the eroded sdK image highlights the blood vessel region (lower values), improving the definition of such region. An erosion with a circular structural element of radio r=2 over the space-directional image was used.

**Fig. 16:**
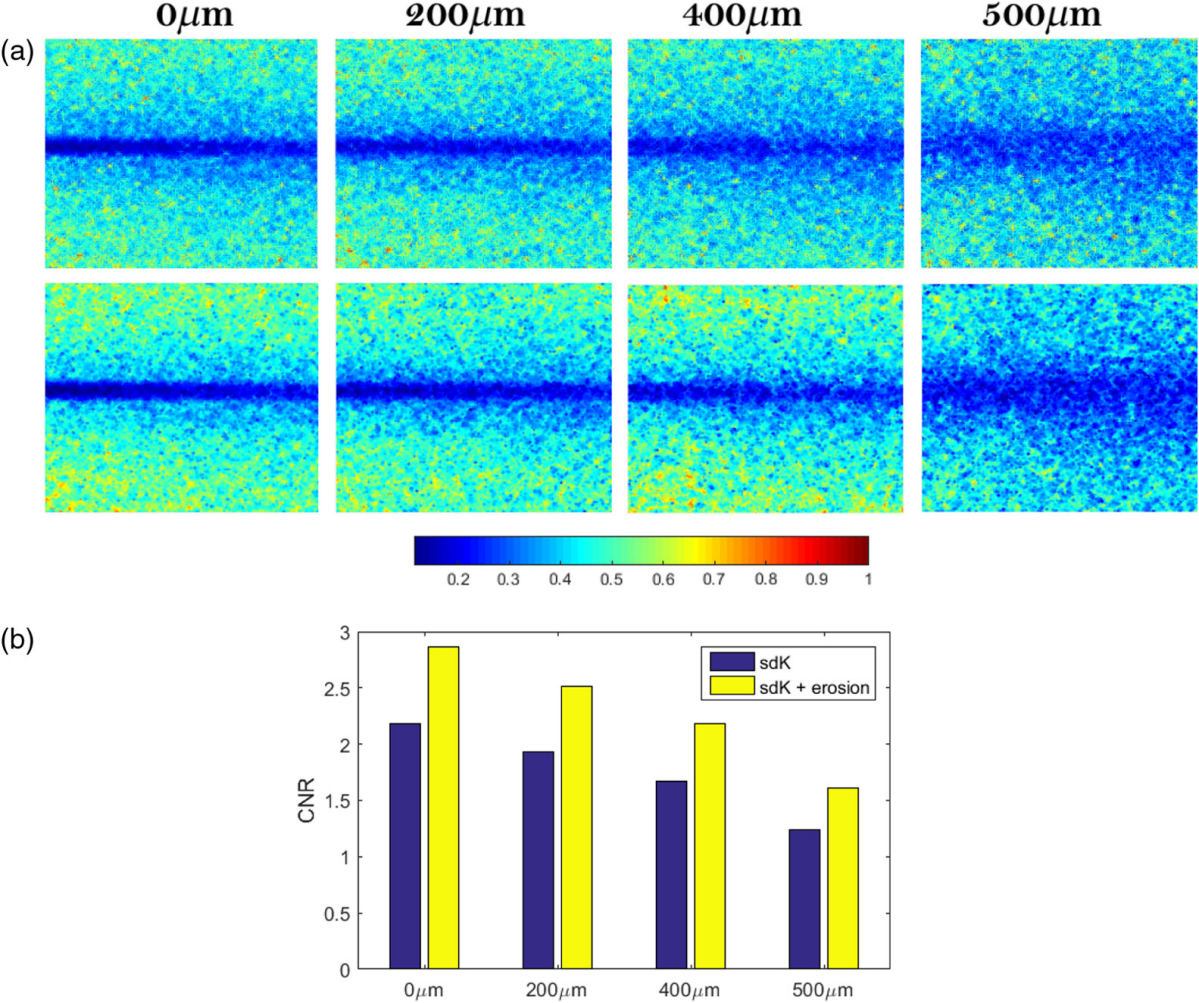
(a) sdK images at different depths before (upper row) and after (bottom row) applying erosion and (b) their corresponding measures of CNR.

**Fig. 17:**
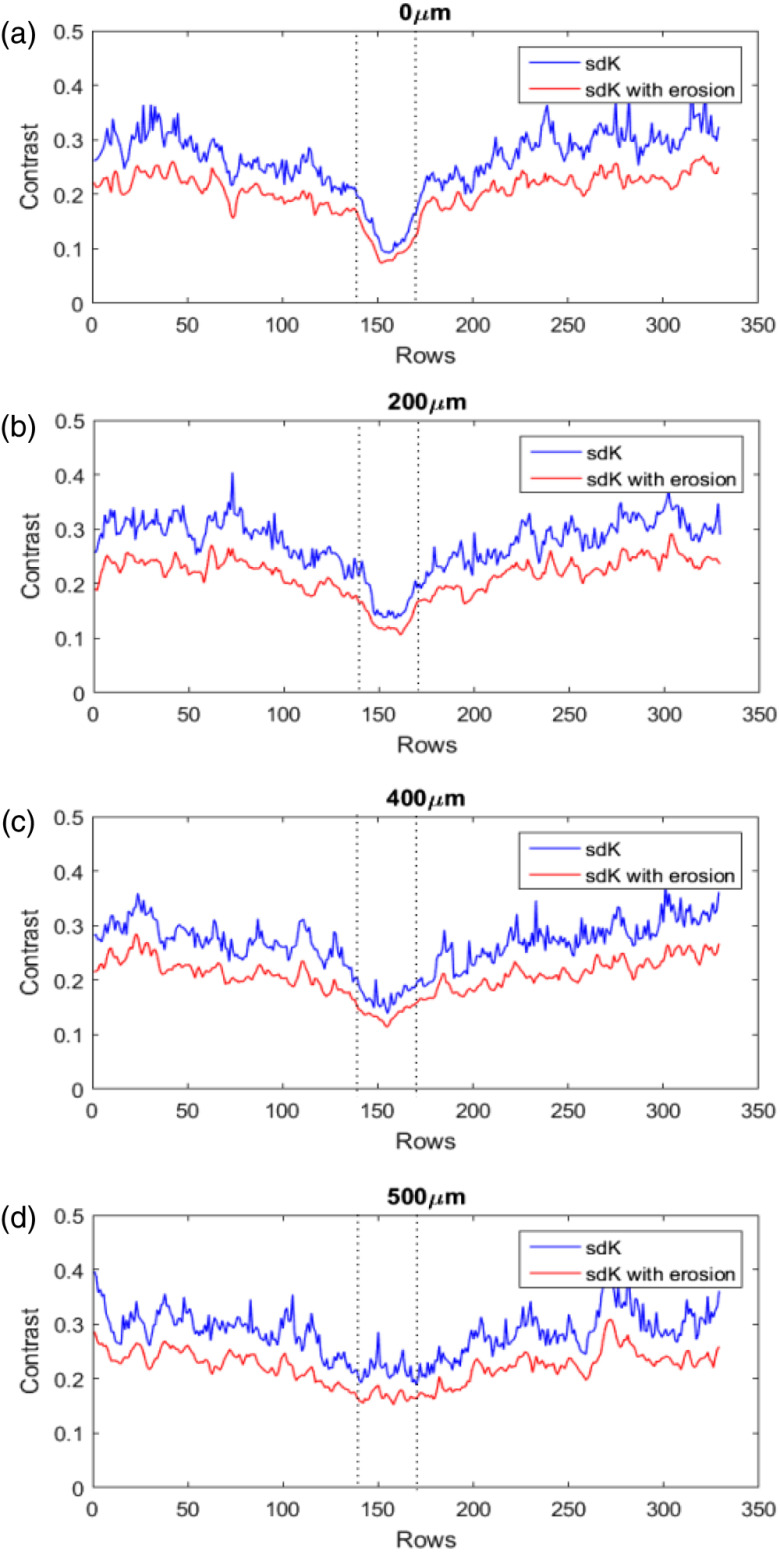
Comparison of the blood vessel profiles processed in Fig. 16. (a) 0  μm, (b) 200  μm, (c) 400  μm, and (d) 500  μm.

Figure 16(a) shows the comparison between the sdK images before (upper row) and after (bottom row) erosion. The difference between static and dynamic regions was highlighted, and the vessel region is better defined. In a general way, the improvement reached by erosion is reflected in the CNR value of Fig. 16(b), where it is observed a significant increment after erosion. If the resulting images of sdK (Fig. 12) are analyzed as unidimensional profiles (blue lines in Fig. 17), the dynamic region (inverted peak between dotted lines) has low variations at 0  μm, which is associated with a high definition of the blood vessel; a similar behavior occurs at 200  μm. However, at 400 and 500  μm, such variations are higher, which generates a low definition. The variations are related to the noise level; at a higher depth, the noise is higher. So, if we consider the blood vessel region as a set of points with near low values affected by a minor set of spurious values (noise), a simple way to remove spurious values is to influence them with the minimum values of its neighborhood (erosion process). As is shown in the red plots of Fig. 17, the dynamic region of sdK is less noisier after it has been eroded improving its visualization. Thus, erosion allows highlighting the vessel even when the depth increases. Since erosion is a postprocessing step, it is not only exclusive for sdK but also it can be used for any other approaches.

## 5 Conclusion

A space-directional approach for contrast computing (sdK) from LSCI images was proposed. This approach emerges as a midpoint of the spatial and temporal LSCI, the two common approaches. The different experiments showed that space-directional contrast is able to reach high noise attenuation while maintaining high temporal resolution. Moreover, using a selection criterion of direction, it was possible to generate a higher distance in value between the static and the dynamic regions, improving visual discrimination between them. Also, it was demonstrated that using a morphological erosion, it was possible to increase the vessel definition since connectivity was improved. As future work, this methodology will be tested at higher depths with a different morphological vessel shape and using images of blood vessels *in vivo*.
